# Prevalence of deep vein thrombosis in acutely admitted ambulatory non-surgical intensive care unit patients

**DOI:** 10.1186/1756-0500-7-431

**Published:** 2014-07-05

**Authors:** Holger Lawall, Ralph Oberacker, Claudia Zemmrich, Peter Bramlage, Curt Diehm, Sebastian M Schellong

**Affiliations:** 1 Department of Angiology, Asklepios Westklinikum Hamburg, Suurheid 20, 22559 Hamburg, Germany; 2 Department of Angiology, Klinikum Karlsbad - Langensteinbach, Karlsbad, Germany; 3 Institut für Pharmakologie und präventive Medizin,, Mahlow, Germany; 4 Krankenhaus Friedrichstadt, Medizinische Klinik II, Dresden, Germany

**Keywords:** Deep vein thrombosis, Prevalence, Ambulatory care, Intensive care, Risk factors, Venous thromboembolism

## Abstract

**Background:**

Data on prevalence rates of venous thromboembolism (VTE) in different patient populations are scarce. Most studies on this topic focus on older patients or patients with malignancies, immobilization or thrombophilia. Less is known about the VTE risk profile of non-surgical patients presenting with a variety of medical diseases of differing severity. Aim of the present study was to investigate VTE prevalence in a pospective cohort study of ambulatory medical intensive care unit patients within 24 h after acute admission.

**Methods:**

Prospective cohort study of 102 consecutive patients after acute admission to medical intensive care unit. Ultrasound compression sonography, APACHE-II-Scoring and laboratory examination was performed within 24 hours after admission.Possible determinants of a high risk of VTE were examined. In all patients with a confirmed diagnosis of DVT or suspicion of PE thoracic computer tomography (CT) was performed.

**Results:**

VTE was found in 7.8% out of 102 of patients, mean APACHE-II-Score was 14 (mortality risk of about 15%). Thrombus location was femoropopliteal in 5 patients, iliacal in 2 and peroneal in 1 patient. Five VTE patients had concomitant PE (62.5% of VTE, 4.9% of all patients). No predictors of prevalent VTE were identified from univariable regression analysis although relative risk was high in patients with a history of smoking (RR 3.40), immobility (RR 2.50), and elevated D-Dimer levels (RR 3.49).

**Conclusions:**

Prevalent VTE and concomitant PE were frequent in acutely admitted ICU patients.

## Background

Venous thromboembolism (VTE) is a common complication not only in surgical but also in acutely ill hospitalized medical patients [1]. A number of risk factors have been identified in medical patients resulting in an increase in VTE rates and mortality. Higher age, cancer, immobilization, infectious disease, and a history of VTE [2-5] are amongst the most important ones, as we know from the large randomized, double-masked, placebo-controlled MEDENOX Study. Multiple logistic regression analysis indicated that these factors were independently associated with VTE in the study population of acute hospitalized patients with heart or respiratory failure, infections, rheumatic disorder or inflammatory bowel disease [6]. Inherited thrombophilia is another established risk factor of VTE. A systematic review and meta-analysis of the prevalence of Factor V Leiden mutation (FVL) and prothrombin mutation (G20210A) (PTM) found those risk factors significantly more often in patients with isolated PE than in controls without VTE (OR 2.06, 95% CI 1.66-2.56, p < 0,0001 for FVL; OR 2.64, 95% CI 1.92-3.63, p < 0.0001 for PTM) [7].

Only a small proportion of those medical at-risk patients are given prophylaxis [2]. Furthermore data show a correlation between mortality and risk score of the patients for DVT [8] and a reduced mortality in case of early thromboprophylaxis [9].

Among medical patients, those admitted to an Intensive Care Unit (ICU) represent a high risk population for VTE [10-13] with a higher prevalence of the aforementioned risk factors. The Asian VOICE study detected a substantial underestimation of VTE risk and non-adherence to guidelines for thromboprophylaxis in medical ICU patients [14]. Additional co-morbidities such as stroke, acute heart failure and respiratory insufficiency are highly prevalent and contribute to a worse outcome [15,16].

Reliable data on the prevalence of VTE in patients acutely admitted to a non-surgical ICU are however not available and we aimed to overcome this lack of data.

## Methods

### Design

This was a prospective survey of daily clinical practice with the prospective inclusion of consecutive ambulatory patients being acutely admitted to the non-surgical intensive-care unit of the Karlsbad-Langensteinbach Hospital in Germany. Ethical approval of the study was not necessary due to the observational nature of the study. Patients provided written informed consent.

### Patient population

Eligible patients had to have an age of at least 18 years and an APACHE-II score of at least 10 which reflects a mortality risk of about 15%. Patients with known or suspected VTE at hospital admission or prior recent hospitalization were excluded. Predominant reasons for admission were acute cardiovascular and pulmonary disorders. Among them were several myocardial infarctions, decompensated heart failure and pneumonias as the ICU focused on patient with acute cardiopulmonary diseases. No mechanically ventilated patient was included.

### Variables obtained

All patients had a clinical workup at hospital admission and the following variables were obtained: reason for admission, APÀCHE-II-score (see below), pharmacotherapy, body weight and height, smoking status, use of compression stockings, co-morbidity, presence of clinical signs of thrombosis (Payr and Homann sign, Lowenberg’s sign, cyanosis, pain, and swelling). The following lab values were determined: leukocytes, thrombocytes, erythrocytes, quick/INR value, partial thromboplastin time, fibrinogen, D-dimere and creatinine.

### Detection of venous thrombo- and pulmonary embolism

**Figure 1:**
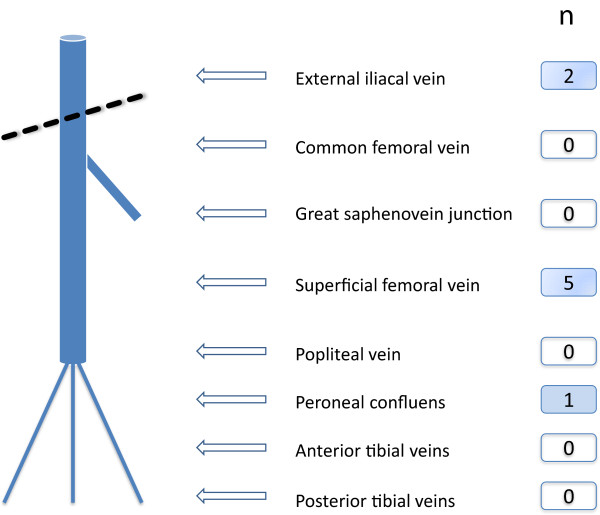
**Localisation of venous thrombosis.***Legend:* n, number of patients with thrombosis at the localisation indicated.

Ultrasound compression sonography (ATL, HDIU 5000, Philips Medical Systems GmbH, Hamburg, Germany) was conducted in all patients within 24 hours after hospital admission according to the EXCLAIM - protocol [17]. Investigators were to scan 10 pre-defined segments of the femoral and popliteal veins including the peroneal and tibial confluens (Figure 1). For the femoral veins, static imaging was performed in the transverse plane with the patient lying supine, while compression was applied at 0.5-1.0 cm intervals along the full length of the vein. Compressed and uncompressed images were obtained at the sites. For the popliteal veins, imaging was performed with the patient in a sitting position with the legs hanging over the edge of the examination table or in case of inability to sit lying supine with the bed being tilted 30% feets downwards, and compressed and uncompressed images were obtained. In all patients with a confirmed diagnosis of DVT or suspicion of PE thoracic computer tomography (CT) was performed.

### APACHE - Score

The APACHE-II (Acute Physiology And Chronic Health Evaluation) score was used to determine the prognosis (mortality) of patients [18-20]. It consists of an Acute Physiologic Score (Table 1), indicating an increased mortality risk with higher values. Age Points are added, reflecting the age dependent increase in mortality. Finally Chronic Health Score points are added for non-surgical patients with pre-existing known organ insufficiency or immunological incompetence.

**Table 1:** APACHE II Scoring

**Acute physiologic score**	**Aberration upwards**					**Aberration downwards**			
**Points**	**+4**	**+3**	**+2**	**+1**	**0**	**+1**	**+2**	**+3**	**+4**
Temperature °C	≥ 41	39 – 40.9		38.5 - 38.9	36 - 38.4	34 - 35.9	32 - 33.9	30 - 31.9	≤ 29.9
MAP mmHg	≥ 160	130 - 159	110 - 129		70 - 109		50 - 69		≤ 49
Heart rate/min	≥ 180	140 - 179	110 - 139		70 - 109		55 - 69	40 - 54	≤ 39
Resp. rate/min	≥ 50	35 - 49		25 - 34	12 - 24	10 - 11	6 - 9		≤ 5
Oxygenation	≥ 500	350 - 499	200 - 349		< 200 | > 70	61 - 70		55 - 60	< 55
Arterial pH	≥ 7.7	7.6 - 7.69		7.5 - 7.59	7.33 - 7.49		7.25 - 7.32	7.15 - 7.24	< 7.15
Sodium	≥ 180	160 - 179	155 - 159	150 - 154	130 - 149		120 - 129	111 - 119	≤ 110
Potassium	≥ 7	6.6 - 6.69		5.5 - 5.59	3.5 - 5.4	3.0 - 3.4	2.5 - 2.9		≤ 2.5
Creatinine mg/dl	≥ 3.5	2.0 - 3.4	1.5 - 1.9		0.6 - 1.4		< 0.6		
Hematocrit %	≥ 60		50 - 59.9	46 - 49.9	30 - 45.9		20 - 29.9		< 20
Leucozytes ×10^3^	≥ 40		20 - 39.9	15 - 19.9	3 - 14.9		1 - 2.9		< 1
Glasgow Coma Scale (GCS)	Points = 15 – actual GCS								
Age points	≤ 44		45 - 54		55 - 64		65 - 74		≥ 75
Points	0		2		3		5		6

### Statistical methods

The analysis of data was performed with the statistical software package SPSS for Windows. Descriptive statistical analyses were performed. Frequencies were reported as means ± standard deviation (SD). Differences were tested by the Chi^2^-test or the Mann–Whitney U-Test for statistical significance using an alpha of 0.05.

## Results

### Patient baseline characteristics

A total of 102 patients, 43 of them women (42.2%) with a mean age of 71.4 ± 11.4 for men and 75.9 ± 14.0 years for women, (p = 0.015 for gender difference) were included. The mean APACHE-II score was 14. Roughly half of the patients were pre-treated with aspirin (47.1%), 18.6% with oral anticoagulation, 13.7% with clopidogrel and 6.9% with any kind of heparin. Further patient characteristics and risk factors are listed in Table 2.

**Table 2:** Patient characteristics and risk factor prevalence in patients with and without DVT

	**Total (n = 102)**	**DVT (+) (n = 8)**	**DVT (-) (n = 94)**
Male gender (n, %)	59 (57.8)	6 (75.0)	53 (56.4)
ASA (n, %)	48 (47.1)	3 (37.5)	45 (47.9)
Clopidogrel (n, %)	14 (13.7)	0	14 (17.9)
Marcumar (n, %)	19 (18.6)	0	19 (20.2)
Heparin (n, %)	7 (6.9)	1 (12.5)	6 (6.4)
Heart failure NYHA ≥ II	68 (66.7)	0	68 (72.3)
Smoker	15 (14.7)	3 (37.5)	12 (12.8)
Immobility* (n, %)	54 (52.9)	6 (75.0)	48 (51.1)
Compressions stockings	14 (13.7)	1 (12.5)	13 (13.8)
Venous insufficiency	30 (29.4)	1 (12.5)	29 (30.9)
Renal insufficiency (n, %)	56 (54.9)	3 (37.5)	53 (56.4)
Malignancy 8 (n, %)	10 (9.8)	1 (12.5)	9 (9.6)
Exsiccosis (n, %)	21 (20.6)	2 (25.0)	19 (20.2)
Diabetes mellitus (n, %)	38 (37.3)	3 (37.5)	35 (37.2)

*Legend:* *Immobility was defined as not able to leave the room but to visit toilet and bath in the room for more than 24 hours before hospital admission, n, number; SD, standard deviation.

### DVT and PE incidence

A previously unknown thrombosis was detected in 8 patients (7.8%), 2 in women and 6 in men. Thrombus location was femoropopliteal in 5 patients, iliacal in 2 and at peroneal confluens in 1 patient. In five out of the eight DVT patients pulmonary embolism (PE) was detected upon CT lung scan (62.5% of DVT patients, 4.9% of all patients). All PE´s were found in patients with a DVT above the knee, twice iliacal and femoropopliteal.

Mean APACHE-II-Score of patients without DVT was 14.56, with DVT 14.38, which was slightly, but statistically not different. Two DVT patients had APACHE-II-Scores of 10 and 11 respectively, the other DVT patients scores of 15, 17, 20 and 21 respectively.

### Risk factors and laboratory values in patients with and without DVT

The following established risk factors for VTE were tested: 1) smoking status, 2) immobility, 3) exsiccosis, 4) diabetes mellitus, 5) coagulation disorders, 6) renal insufficiency (Table 3). While there was a nominally increased relative risk in those with smoking (RR 3.40), immobility (RR 2.50), exsiccosis (RR 12.9) and diabetes (RR 1.20) and a decreased risk in those with renal insufficiency (RR 0.49), confidence intervals were too wide and only smoking became borderline significant (p = 0.06).

**Table 3:** Relative risk of traditional risk factors and laboratory results in patients with vs. without DVT

	**RR [95% CI]**	**P - value**
**Risk factors**		
Smoker (vs. nonsmoker)	3.40 (0.93-13.06)	0.06
Immobility (yes vs. no)	2.50 (0.53-11.79)	0.23
Exsiccosis (yes vs. no)	1.29 (0.28-5.92)	0.79
Diabetes mellitus (yes vs. no)	1.20 (0.29-5.09)	0.81
Renal insufficiency (yes vs. no)	0.49 (0.12-1.95)	0.30
**Laboratory results**		
D-dimere (increased vs. normal)	3.49 (0.89-13.71)	0.58
CRP (increased vs. normal)	1.97 (0.42-9.27)	0.38
Leukocytes (increased vs. normal)	1.95 (0.49-7.73)	0.33
Fibrinogen (increased vs. normal)	1.5 (0.39-5.92)	0.55
PTT (increased vs. normal)	1.37 (0.30-6.27)	0.69
Erythrocytes (increased vs. normal)	0.17 (0.02-1.31)	0.05
Creatinine (increased vs. normal)	1.15 (0.29-4.53)	0.84
Thrombocytes (increased vs. normal)	1.03 (1.03-1.17)	0.28
Quick (decreased vs. normal)	0.44 (0.06-3.41)	0.41

The following laboratory values were tested: D-dimere, leucocytes, C-reactive protein (CRP), partial thromboplastin time (PTT), fibrinogen, thrombocytes, erythrocytes, quick, creatinin. No significant correlation was found for any of the named laboratory values. The highest risk ratios were found for D-dimere (RR 3.49), CRP (RR 1.97) and leucocytes (RR 1.95).

### Clinical follow up

6 out of 8 DVT patients (75%) were transferred from the ICU to a general ward within a week after admission. One DVT patient stayed at the intensive care unit for 14 days and 1 patient died due to sepsis.

## Discussion

The present prospective analysis is the first to report the prevalence of DVT in acutely admitted non-surgical intensive care unit patients in Germany. We investigated the isolated prevalence rate within 24 hours after hospital admission not considering DVT incidence rates during the ICU stay. We found a DVT prevalence of 7.8% with two thirds of the DVT positive patients presenting additional pulmonary embolism. The data are limited however by the patient number that was found to be too low to conduct a proper predictor analysis. This calls for a larger prospective survey to be conducted in this high risk patient group.

### Prevalence of deep venous thrombosis

Limited data are available comparing VTE prevalence in critical ill medical patients admitted to non-surgical intensive care units with a variety of diseases, but not mechanically ventilated. Nevertheless these patients are frequent and show an increased mortality risk. In general medical units Lawall et al. [21] found in not critically ill patients a prevalence of 2.6% in medical patients, and Cheng [22] in a very similar patient population a prevalence of 1.7%. Oger et al. [23] reported (within 48 hours of hospital admission, including incidence during hospitalization, without suspected VTE at admission) an incidence of 5.5% (95% CI 3.1-9.5) asymptomatic DVT using compression ultrasound and 17.8% (95% CI 0.0-12.7) among patients over 80 years. A review by Crowther and Cook comprised data of several studies and found DVT in 5 – 10% of critically ill patients even if they receive unfractionated heparin for prophylaxis [24]. The majority appeared clinically silent, a known phenomenon from earlier studies [25-27]. One prospective cohort study of twice weekly ultrasounds identified proximal DVT during ICU stay in 25 out of 261 (9.6%) patients [28]. The PROTECT trial tested dalteparin versus unfractionated heparin and found at intensive care unit patients with a mean APACHE-II-Score of 21.5 a prevalence during ICU stay of 5.5% [29]. This number represents a cumulative prevalence and incidence in patients with ongoing thromboprophylaxis. Hong et al. prospectively screened 90 ICU patients without prophylaxis and repeated admission ultrasound 5–7 days after admission and 11.1% of patients developed DVT [30]. Sud et al. [13] compared ultrasound screening versus clinically suspected DVT case finding in medical-surgical ICU patients and found 85 proximal DVT´s per 1000 patients. These numbers are comparable to our findings.

Wells et al. found a high rate of false positive results with careful clinical assessment for DVT only or in combination with inflammation markers. They concluded that this approach for DVT screening is not useful in this patient group alone [31].

Older data reported by Hirsch et al. [25] for medical ICU patients reported a DVT prevalence of 33%, but this number included 8 months follow-up with ultrasound screenings after discharge from MICU.

### Prevalence of pulmonary embolism

We found accompanying pulmonary embolism (PE) in 5 patients (62.5% of DVT patients, 4.9% of all patients). This rate is higher than in the literature described. There are data showing silent PE in around 33% of DVT patients for instance in the previously mentioned study by Lawall et al. in internal medicine and in a recent study by Tzoran et al., who found in 33% of DVT patients additional PE [32]. But those patients were no ICU patients, which are at higher risk for DVT than general medical ward patients. A large prospective multicenter Chinese trial found a prevalence of DVT during medical ICU stay including a 90 day follow up ultrasound and clinical examination of 7.3% with additional PE in 0.5% and isolated PE in 2.1% [33]. Berlot et al. retrospectively reviewed 600 autopsies and clinical data of all patients who died at a mixed surgical and medical ICU between 1996 and 2007 [34]. All patients received prophylaxis with subcutaneous low-molecular weight heparin. They found 13 confirmed PE, 20 not confirmed and 73 PE´s only discovered at the autopsy. The overall incidence was 14.3%. Among all non-surgical co-morbidities the presence of acute renal failure was associated with a higher risk of missed diagnoses, which was also found with a higher frequency in cases of septic shock.

### Predictors of deep venous thrombosis

Several risk factors have been shown to be associated with DVT. We found a tendency towards an increased DVT risk for smoker, immobile, exsiccotic and diabetic patients, but none reaching statistical significance. No correlation was found in our study population for the established risk factors age and gender or for laboratory values. D-Dimer, leucocytes and CRP values showed a tendency towards an increased risk. Unfortunately those values are often increased anyway at hospital admission resulting from the primary admission diagnosis. They represent an ongoing unspecified inflammation process, which is common in this patient population also without a present DVT. A clinical use as DVT predicting risk factor cannot be deduced from those results. No correlation between DVT prevalence and a high APACHE-II-Score was found, but the incidence of DVT is quiet low for interpretation of statistical correlations.

### Limitations

The patient group under investigation reflects a heterogeneous medical patient group with a wide range of differing thromboembolic risk constellations, predominantly acute cardiovascular and pulmonary disorders without the need for mechanical ventilation The strength of the study is to have searched for predictors characterizing thromboembolic risk in these patients, who are frequently treated at ICUs but not extensively studied so far regarding thrombosis prevalence. As the patient numbers and resulting DVT/PE prevalence rates were too low, no significant predictors were identified. A larger survey with higher numbers of DVT and PE patients is clearly needed to allow a better patient characterisation using easy – available clinical signs and laboratory values.

## Conclusions

Our results show a rather high asymptomatic DVT prevalence in acutely ill medical ICU admitted patients with APACHE-II-score > 10 and a considerable amount of accompanying pulmonary embolism. The studied patient group nonetheless lacks clear thromboembolic risk characterisation as conventional risk factors, laboratory values or the value of the APACHE-II-score seem to be not sufficient. This result calls for further investigation of a larger cohort of this special patient group. As long as data are missing physicians in this clinical setting should be aware of thromboembolic complications and consider a general DVT screening at hospital admission for this patient population.

## Competing interests

The authors report no conflict of interests.

## Authors’ contributions

All authors made substantial contributions to conception and design, or acquisition of data, or analysis and interpretation of data. HL and CD designed the study, RA acquired the data, CZ and PB analysed the data and drafted the manuscript. The other authors revised the manuscript for important intellectual content and all authors granted final approval of the manuscript to be published.
